# Genetic markers of enhanced functional antibody responses to COVID-19 vaccination

**DOI:** 10.1016/j.vaccine.2025.127379

**Published:** 2025-06-16

**Authors:** Ruth A. Purcell, L. Carissa Aurelia, Lilith F. Allen, Katherine A. Bond, Deborah A. Williamson, Janine M. Trevillyan, Jason A. Trubiano, Bruce D. Wines, P. Mark Hogarth, Jennifer A. Juno, Adam K. Wheatley, Thi H.O. Nguyen, Kanta Subbarao, Katherine Kedzierska, Stephen J. Kent, Siddhartha Mahanty, Kevin John Selva, Amy W. Chung

**Affiliations:** a Department of Microbiology and Immunology, The Peter Doherty Institute for Infection and Immunity, University of Melbourne, Melbourne, VIC 3000, Australia; b Victorian Infectious Diseases Reference Laboratory (VIDRL), The Peter Doherty Institute for Infection and Immunity, Melbourne, VIC 3000, Australia; c Department of Infectious Diseases, The Peter Doherty Institute for Infection and Immunity, University of Melbourne, Melbourne, VIC 3000, Australia; d School of Medicine, University of St Andrews, Fife, KY16 9TF, Scotland; e Centre for Antibiotic Allergy and Research, Austin Health, Heidelberg, VIC 3084, Australia; f Department of Medicine, University of Melbourne, Parkville, VIC 3010, Australia; g Department of Infectious Diseases, Peter MacCallum Cancer Centre, Melbourne, VIC 3000, Australia; h National Centre for Infections in Cancer, Peter MacCallum Cancer Centre, Melbourne, VIC 3000, Australia; i Immune Therapies Group, Burnet Institute, Melbourne, VIC 3004, Australia; j Department of Immunology and Pathology, Central Clinical School, Monash University, Melbourne, VIC 3004, Australia; k Department of Clinical Pathology, University of Melbourne, Melbourne, VIC 3000, Australia; l WHO Collaborating Centre for Reference and Research on Influenza, The Peter Doherty Institute for Infection and Immunity, University of Melbourne, Melbourne, VIC 3000, Australia; m Melbourne Sexual Health Centre and Department of Infectious Diseases, Alfred Health, Central Clinical School, Monash University, Melbourne, VIC 3004, Australia

**Keywords:** IgG Allotype, FcγR polymorphism, Fc functions, Immunogenetics, Vaccine, SARS-CoV-2

## Abstract

**Introduction:**

Substantial population-level variation in vaccine-specific antibody responses has been observed following global coronavirus disease 2019 (COVID-19) vaccination efforts. Beyond the influence of clinical and demographic features, immunogenetic variation is suggested to underlie divergent serological responses following COVID-19 vaccination of distinct populations.

**Methods:**

Immunoglobulin G1 (IgG1) allotypic markers (G1m) for 121 COVID-19 vaccinated healthy adults were genotyped via Sanger sequencing. Vaccine-specific IgG and Fc gamma receptor (FcγR) engagement were characterised via bead-based multiplex array.

**Results:**

Following two COVID-19 vaccine doses, G1m1,17^+/+^ compared to G1m-1,3^+/+^ vaccinees had increased IgG and FcγR engagement specific for the antigenically conserved SARS-CoV-2 Spike 2 (S2) domain. IgG targeting antigenically novel SARS-CoV-2 receptor binding domain (RBD) trended higher in G1m1,17^+/+^ vaccinees, facilitating increased RBD-specific FcγR2a-R131 and FcγR2b binding.

**Conclusion:**

Primary COVID-19 vaccination induced increased S2-specific IgG in G1m1,17^+/+^ vaccinees, facilitating enhanced anti-viral FcγR engagement and suggesting immunogenetics may be a valuble consideration for next-generation vaccine design.

## 1. Introduction

The immunogenicity of vaccines against coronavirus disease 2019 (COVID-19) is highly heterogenous within and between diverse populations, regardless of vaccine platform [1–4]. Clinical and demographic features, such as chronic comorbidities [3] and older age [4], have been associated with impaired antibody responses following COVID-19 vaccination. However, the potential influence of immunogenetics upon vaccine immunogenicity remains underexplored [5,6]. Single nucleotide polymorphisms within immunoglobulin G (IgG) heavy chain constant regions, termed IgG allotypes, have previously been associated with modulation of antigen-specific antibody responses following infection with or vaccination against a range of viral, bacterial, and parasitic pathogens [6–12]. Given the geographic clustering of allotypes resulting from a Mendelian inheritance pattern, these genetic markers may inform population-specific vaccination strategies [6].

Beyond neutralising antibody titres—the best described correlate of protection against COVID-19 [13]—other humoral immune responses, including non-neutralising antibodies show considerable variation between vaccinees [1–4]. In contrast to neutralisation, anti-viral Fc effector functions are more durable and better maintained against viral variants since non-neutralising antibodies targeting antigenically conserved regions can facilitate cross-protective FcγR activation [14–16]. As such, understanding potential genetic determinants of improved functional antibody responses may inform optimisation of next-generation vaccination strategies. IgG allotypes are proposed to influence IgG1–4 subclass distribution such that the balance of highly cytophilic subclasses (IgG1 and IgG3) and subclasses with reduced Fc gamma receptor (FcγR) affinities (IgG2 and IgG4) is altered [17]. Consequently, increased proportions of cytophilic IgG have the potential to enhance FcγR binding and downstream Fc effector functions induced by non-neutralising antibodies [18]. Furthermore, amino acid variation within the antibody Fc region has been reported to influence IgG affinity for FcγRs [19,20].

Here, within a cohort of COVID-19 vaccinated healthy adults who received primary mRNA-based BNT162b2 or adenoviral vector-based AZD1222 plus mRNA booster, we assessed the influence of Immunoglobulin Heavy Constant Gamma 1 (*IGHG1*) genetic variation upon severe acute respiratory syndrome coronavirus 2 (SARS-CoV-2)-specific IgG levels and FcγR engagement. G1m1,17/G1m1,17 homozygosity was associated with increased IgG and correspondingly increased antibody-mediated FcγR binding specific for the antigenically conserved spike 2 (S2) domain. G1m1,17-associated increases in functional antibody responses against antigenically novel epitopes were less pronounced.

## 2. Methods

### 2.1. Study participants and sample collection

SARS-CoV-2 vaccine plasma, granulocyte, and PBMC samples were collected from 121 vaccinees 1 month post-second dose BNT162b2 (Pfizer-BioNTech) or AZD1222 (AstraZeneca) vaccination and 1 month post-mRNA booster (BNT162b2 or mRNA-1273 (Moderna)), as previously described [14,21–23]. Vaccinee cohort demographics are described in Table 1. Study protocols were approved by the University of Melbourne (#2056689, #20734, #21560, #21626, and #13344), Austin Health (#HREC/73256/Austin-2021), and Melbourne Health (HREC/63096/MH-2020 and HREC/68355/MH-2020).

### 2.2. DNA extraction, Polymerase chain reaction (PCR), and Sequencing

G1m1/G1m-1 and G1m3/G1m17 typing of study participants was performed via PCR and Sanger sequencing, as previously described [21,24]. Briefly, genomic DNA was extracted from granulocytes or PBMCs using the QIAamp DNA Blood Mini Kit (Qiagen GmbH, Hilden, Germany; 51104) according to the manufacturer’s instructions. Amplification of the human C_H_1 and C_H_3 domains of *IGHG1* was performed using the AccuPrime *Taq* DNA Polymerase, High Fidelity system (Thermo Fisher Scientific, 12346094). Dual direction sequencing of PCR products was performed by the Australian Genome Research Facility (AGRF, Melbourne, Australia). Geneious Prime version 2023.2.1 was used for sequence analysis and genotypes were manually called.

### 2.3. Bead-based multiplex assay

To characterise antigen-specific plasma antibody responses, we utilized a customised SARS-CoV-2 antigen multiplex bead array, validated as previously described [14,21–23]. Briefly, plasma diluted in PBS at an appropriate single concentration (1:100 for dose 2 AZD1222 vaccinee plasma, and 1:800 for dose 2 BNT162b2 vaccinee plasma and all mRNA booster vaccinee plasma) were incubated with antigen-coupled beads (Supplementary Table 1) and the levels of total IgG, IgG1–4 or soluble FcγR dimer engagement (Supplementary Table 2) were assessed via FLEXMAP 3D (Luminex, Austin, TX, USA). Optimal plasma dilutions were determined by calculating the average EC_50_ response specific to each vaccine regimen timepoint and selected for suitability across detectors (Supplementary Fig. 1). Anti-IgG and anti-IgG1 antibody detector clones were selected following demonstration of equivalent binding to the different IgG1 allotypes of interest [21]. Detailed methods are described in Supplementary materials.

### 2.4. Statistical analysis

Prism GraphPad version 10.4.0 (GraphPad Software, San Diego, CA, USA) was used to develop graphs and perform the statistical analyses described in the figure legends.

## 3. Results

### 3.1. G1m1,17 haplotype associates with increased IgG against SARS-CoV-2 spike 2 domain

**Fig. 1.**
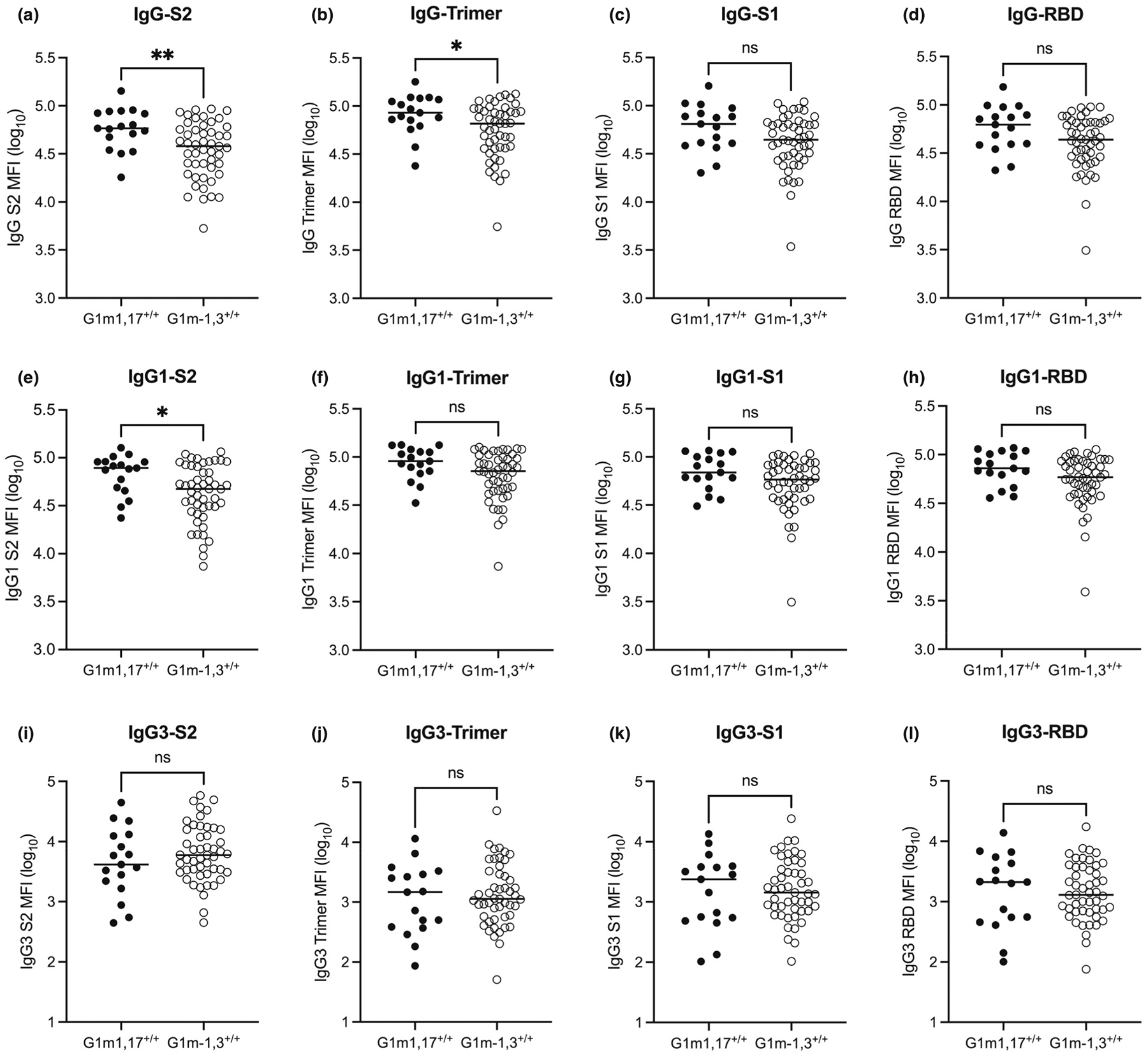
Association of *IGHG1* haplotypes with SARS-CoV-2-specific antibody levels following two COVID-19 vaccine doses**. (a)** S2-, **(b)** Trimer-, **(c)** S1-, and **(d)** RBD-specific IgG levels. **(e)** S2-, **(f)** Trimer-, **(g)** S1-, and **(h)** RBD- specific IgG1 levels. **(i)** S2-, **(j)** Trimer-, **(k)** S1-, and **(l)** RBD- specific IgG3 levels. Mann-Whitney *U* tests performed between G1m1,17/G1m1,17 and G1m-1,3/G1m-1,3 vaccinees for each antibody-antigen feature. *P* < 0.01 (**); *P* < 0.05 (*); non-significant (ns).

We identified five *IGHG1* haplotypes at expected frequencies within our COVID-19 vaccinated cohort (Table 1). Total IgG and IgG1–4 subclass levels against whole SARS-CoV-2 spike trimer (ST) as well as S2, spike 1 (S1) domain, and receptor binding domain (RBD)—distinct subunits of ST—were then assessed. Importantly, the degree of antigenic conservation with seasonal coronaviruses varies between ST subunits, with S2 being most conserved and RBD being most novel [25]. Following two COVID-19 vaccine doses, G1m1,17/G1m1,17 vaccinees, as compared to G1m-1,3/G1m-1,3 vaccinees, generated increased total IgG against SARS-CoV-2 S2 and ST (Fig. 1a–b) as well as increased S2-specific IgG1 (Fig. 1e). Although not statistically significant, S1- and RBD-specific total IgG as well as ST-, S1- and RBD- specific IgG1 levels trended higher in G1m1,17/G1m1,17 vaccinees (Fig. 1c–d, 1f–h). These data suggest that the influence of IgG1 allotypes upon COVID-19 vaccine responses is antigen-dependent, with the effect being most pronounced in the context of IgG targeting antigenically conserved epitopes.

Upon multiple comparisons between all five *IGHG1* haplotypes, we observed an intermediate anti-S2 IgG response for heterozygotes, albeit with reduced statistical power comparing all haplotypes (Supplementary Fig. 2). Following a third COVID-19 vaccine dose, the influence of G1m1,17 homozygosity upon elevated S2-specific IgG was diminished (Supplementary Fig. 3).

Using the anti-IgG3 detector clone MTG34, no association between *IGHG1* haplotype and quantity of SARS-CoV-2-specific IgG3—the second most dominant IgG subclass following two BNT162b2 or AZD1222 doses [26,27]—was observed (Fig. 1i–l). As we were unable to identify an IgG3-specific detection antibody that was able to equivalently bind IgG3 allotypes of interest (Supplementary Fig. 4), we were unable to assess the potential influence of *IGHG3* haplotypes upon SARS-CoV-2-specific IgG responses.

### 3.2. Elevated S2-specific IgG facilitates increased FcγR engagement by G1m1,17 homozygous vaccinees

**Fig. 2.**
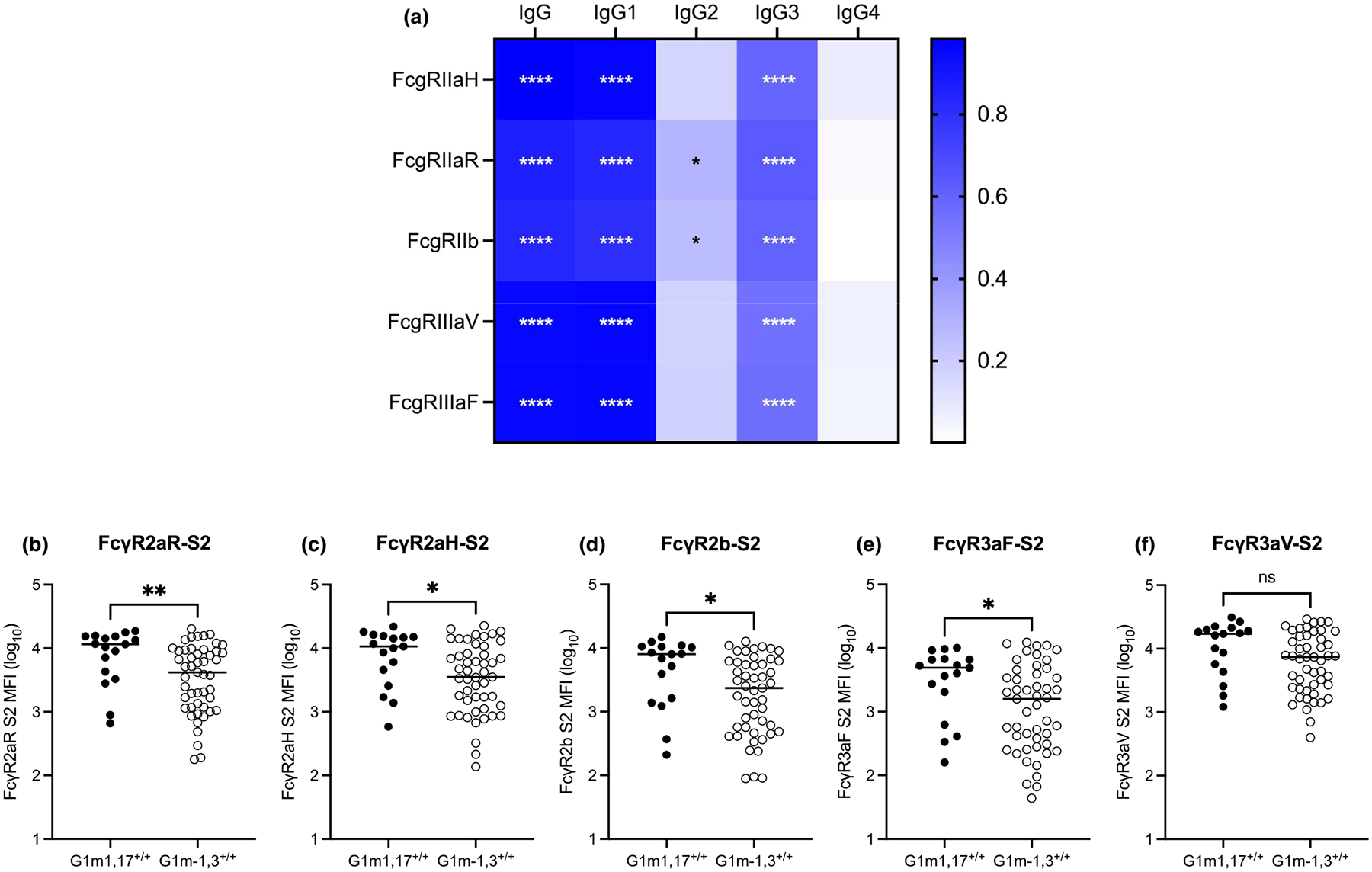
Increased S2-specific IgG levels drive increased S2-specifc FcγR engagement in an FcγR type- and polymorphism- dependent manner. **(a)** Heatmap of spearman correlation coefficients calculated for associations between S2-specifc FcγR engagement and S2-specifc total IgG or IgG subclass levels following two COVID-19 vaccine doses. S2-specifc **(b)** FcγR2aR, **(c)** FcγR2aH, **(d)** FcγR2b, **(e)** FcγR3aF, and **(f)** FcγR3aV engagement following two COVID-19 vaccine doses. Mann-Whitney *U* tests performed between G1m1,17/G1m1,17 and G1m-1,3/G1m-1,3 vaccinees for each S2-specific FcγR response. *P* < 0.0001 (****); *P* < 0.01 (**); *P* < 0.05 (*); non-significant (ns).

Of the four IgG subclasses, IgG1 and IgG3 possess the highest affinities for FcγRs [28]. This, coupled with the IgG1-dominant response following two COVID-19 vaccine doses [26,27], underpinned strong correlation of S2-specific total IgG and IgG1 levels with FcγR engagement (Fig. 2a). Consequently, the increased anti-S2 IgG, specifically IgG1, observed in G1m1,17/G1m1,17, as compared to G1m-1,3/G1m-1,3, vaccinees facilitated twofold to threefold increased FcγR2 and FcγR3 binding (Fig. 2b–f), although the statistical significance of this effect appeared to be Fc receptor type- and polymorphism- specific.

IgG allotypic variation has been associated with differential IgG-FcγR binding and FcγR activation [19,20]. Previous studies suggest that G1m1,17 IgG1 may bind the FcγR2a-R131 polymorphism with higher affinity than does G1m-1,3 IgG1 [19]. Using biolayer interferometry (BLI), we also observed that the apparent equilibrium dissociation constants (K_D_s) of G1m1,17 IgG1 binding to FcγR2aR and FcγR2b, but not to FcγR2aH, suggested a higher affinity interaction as compared to G1m-1,3 IgG1 (Supplementary Fig. 5). For the more antigenically novel SARS-CoV-2 antigens S1 and RBD, as well as for ST, significantly increased antibody-mediated FcγR engagement by G1m1,17/G1m1,17 vaccinees was only observed for FcγR2aR and inhibitory FcγR2b, though engagement of other FcγRs trended higher in G1m1,17/G1m1,17 vaccinees (Supplementary Fig. 6). Thus, along with differential vaccine-induced IgG subclass titres, *IGHG1* haplotype-specific differences in IgG1-FcγR binding to FcγR polymorphisms may contribute towards variable FcγR engagement.

As predicted by anti-S2 IgG responses, FcγR binding by G1m1,17/G1m-1,3 heterozygous vaccinees represented an intermediate, though not significantly different, response (Supplementary Fig. 7). No significant differences in FcγR binding were observed between *IGHG1* haplotypes following dose 3 (Supplementary Fig. 3).

## 4. Discussion

In an Australian cohort of COVID-19 naïve healthy adults who received primary BNT162b2 or AZD1222 vaccination, we demonstrated that G1m1,17/G1m1,17 vaccinees generated significantly increased IgG and IgG1 against S2, the most conserved SARS-CoV-2 spike subunit. Responses against more novel antigens such as the RBD trended slightly higher in G1m1,17/G1m1,17 vaccinees. These findings suggest that G1m1,17 haplotype-associated increases in SARS-CoV-2-specific IgG responses may be dependent upon elevated levels of pre-existing humoral immune memory to conserved epitopes. We hypothesise that when cross-reactive responses are mounted against an antigen for which prior immune memory exists, for example in this case, against antigenically conserved seasonal coronavirus epitopes, G1m1,17/G1m1,17 vaccinees may have a small but significant advantage in generating a heightened recall antibody response.

However, since trends of elevated S2-specifiic IgG in G1m1,17/G1m1,17 individuals following three COVID-19 vaccines were not statistically significant, G1m haplotypes do not appear to modulate maximal vaccine-specific IgG titres. As such, G1m1,17/G1m1,17 vaccinees may only be advantaged in the setting of boosting a significantly waned memory response (i.e. cross-reactive S2 titres upon primary COVID-19 vaccination) prior to achieving maximal SARS-CoV-2 antigen-specific antibody titres (i.e. responses following COVID-19 booster vaccination [29]).

Since antigen-specific IgG1 and IgG3 titres are strongly correlated with FcγR2 and FcγR3 binding, we expected that the increased S2-specific total IgG and IgG1 levels observed in G1m1,17/G1m1,17 vaccinees may translate to broadly increased engagement of low-affinity FcγRs. However, the extent of the G1m1,17-associated increase in FcγR engagement was dependent upon antigen as well as FcγR type and polymorphism. Significantly increased S2-specific FcγR engagement in G1m1,17/G1m1,17 vaccinees was observed for all tested FcγRs except the higher-affinity FcγR3a polymorphism FcγR3aV. Furthermore, FcγR2aR and FcγR2b were the only FcγRs for which significantly increased ST-, S1-, and RBD- specific FcγR engagement were observed in G1m1,17/G1m1,17 vaccinees. These data suggest that FcγR2a and FcγR3a genetic variation may further influence the magnitude of the Fc functional enhancement underpinned by increased IgG in G1m1,17/G1m1,17 vaccinees.

In agreement with our BLI data, previous data suggest that G1m1,17 IgG1 binds the FcγR2aR polymorphic FcγR2a variant with higher affinity than does G1m-1,3 IgG1 [19]. In prior studies, this effect was not observed for FcγR2aH, FcγR3aV, and FcγR3aF—other low-affinity activating FcγRs—or for high-affinity FcγRI [19]. These data suggest a potential epistatic interaction between the *IGHG1* haplotype G1m1,17 and the lower-affinity FcγR2a polymorphism, FcγR2aR, whereby a combination of these two genotypes may promote increased FcγR2a-driven enhancement of anti-viral Fc effector functions. However, given that G1m1,17/G1m1,17 vaccinees also demonstrated increased binding to inhibitory receptor FcγR2b, their potential for increased FcγR2aR-mediated effector functions may be overridden. Future studies should confirm the functional consequences of increased FcγR2aR engagement by G1m1,17 IgG1 using cell-based Fc functional assays employing effector cells with known FcγR polymorphisms.

Our study is limited by several technical and cohort-specific restrictions. Given that *IGHG1* and *IGHG3* are in linkage disequilibrium and tend to be inherited as haplotype blocks, IgG1 and IgG3 allotypes have been associated with modulation of antigen-specific levels of the alternate IgG subclass, as well as total antigen-specific IgG [7,9,10]. However, as we were unable to identify an anti-IgG3 detector clone that equivalently binds IgG3 allotypes of interest, the influence of IgG3 allotypes, and by extension, IgG1 allotypes, upon IgG3 levels could not be determined reliably. The low frequency of the homozygous G1m1,17/G1m1,17 genotype posed a substantial challenge to recruiting the number of vaccinees required to perform sufficiently powered statistical calculations. This limitation was especially evident at the dose 3 time point for which genotyping identified only 14 G1m1,17/G1m1,17 participants. Given that the Mendelian inheritance pattern of IgG haplotypes facilitates a geographic clustering of genotypes [6], future studies exploring the influence of IgG allotypes upon antibody responses would benefit from multi-centre international cohorts recruited from the specific geographical regions in which the haplotypes of interest are most prevalent.

IgG haplotype-associated differences in antigen-specific Ig titres, Fc functional antibody responses, and protection against disease have been reported for a range of viral, bacterial, and parasitic pathogens [7–12,17]. While the influence of distinct haplotypes appears to be pathogen-specific [6], the G1m1,17 haplotype has consistently been associated with elevated antibody titres against viral antigens [9,17]. Here, following two COVID-19 vaccine doses, we demonstrated that the G1m1,17 haplotype is associated with a small but significant increase in IgG titres and FcγR engagement, predominantly targeting the antigenically conserved S2 antigen. Larger independent cohort studies of vaccination against COVID-19 and other infectious diseases are required to determine if these IgG haplotype-associated differences in functional antibody responses contribute to efficacy of primary vaccination.

## Supplementary Material

1

## Figures and Tables

**Table 1:** Cohort demographics.

	Dose 2	Dose 3 (mRNA)
**Number of Vaccinees (*n*)**	121	84
**Age (mean (range))**	43 (20–82)	43 (22–82)
**Female (*n* [%])**	78 (64)	58 (69)
**Primary Vaccine type**		
AZD1222 (*n*)	42	26
BNT162b2 *(n)*	79	58
Days p.v. (median (IQR))	30 (20–32)	30 (28–34)
**G1m haplotype**		
G1m1,17/G1m1,17 (*n*)	17	14
Age (mean (range))	43 (24–68)	46 (29–69)
G1m-1,3/G1m-1,3 (*n*)	49	32
Age (mean (range))	44 (20–82)	45 (25–82)
G1m1,17/G1m-1,3 (*n*)	41	27
Age (mean (range))	44.5 (22–68)	43 (22–67)
G1m1,17/G1m1,3 (*n*)	6	5
Age (mean (range))	34 (29–43)	35 (29–44)
G1m1,3/G1m1,3 (*n*)	8	6
Age (mean (range))	34 (23–50)	35 (23–50)

IQR, Interquartile range; p.v., post-vaccination.

## Data Availability

Data will be made available on request.

## Appendix A. Supplementary data

Supplementary data to this article can be found online at https://doi.org/10.1016/j.vaccine.2025.127379.
